# Fipronil Degradation in Soil by *Enterobacter chengduensis* Strain G2.8: Metabolic Perspective

**DOI:** 10.3390/life13091935

**Published:** 2023-09-20

**Authors:** Caio Prado, Rodrigo Pereira, Lucia Durrant, Rômulo Júnior, Francine Piubeli, Maricy Bonfá

**Affiliations:** 1 Engineering School of Lorena, University of São Paulo, Lorena 12602-810, Brazil; caioachiles@usp.br; 2 Faculty of Biological and Environmental Sciences, Universidade Federal da Grande Dourados, Dourados 79825-070, Brazil; rodrigopereira@ufgd.edu.br; 3 BioSage, Lawreceville, NJ 08648, USA; durrantclean@gmail.com; 4 Embrapa Agropecuária Oeste, Dourados 79804-970, Brazil; romulo.scorza@embrapa.br; 5 Pharmacy Faculty, University of Seville, 41012 Seville, Spain; piubeli@us.es

**Keywords:** pesticide, bioprospecting, metabolites, biodegradation

## Abstract

Fipronil is an insecticide widely used in the agricultural and veterinary sectors for its efficacy in pest control. The presence of fipronil in the environment is mainly due to agricultural and domestic practices and is frequently found in different types of environmental matrices in concentrations ranging from µg/L to mg/L and can be hazardous to non-target organisms due to its high toxicity. This study was carried out to obtain and characterize microorganisms from soil which are capable of biodegrading fipronil that could be of great biotechnological interest. For this purpose, bioprospecting was carried out using fipronil (0.6 g/L) as the main source of carbon and nitrogen for growth. Once obtained, the strain was identified by sequencing the 16S ribosomal RNA (rRNA) gene and the capacity to degrade fipronil was monitored by GC-MS. Our study showed a presence in soil samples of the strain identified as *Enterobacter chengduensis*, which was able to metabolize fipronil and its metabolites during the mineralization process. *Enterobacter chengduensis* was able to biodegrade fipronil (96%) and its metabolites fipronil-sulfone (92%) and fipronil-sulfide (79%) in 14 days. Overall, the results of this study provided a bacterium with great potential that could contribute to the degradation of fipronil in the environment.

## 1. Introduction

Fipronil is an insecticide widely used in the agricultural and veterinary sectors due to its efficacy in pest control [1]. This compound, which belongs to the phenylpyrazole group of insecticides, acts directly on the target nervous system, blocking the gamma-aminobutyric acid (GABA) receptors and altering the permeability of the chloride ion flux, causing paralysis and the death of insects [2]. Due to its potential for action, according to data from the Brazilian Institute of Environment and Renewable Natural Resources (IBAMA), it is estimated that in 2020 alone more than 2000 tons of fipronil were commercialized [3]. Although this compound is highly effective against insects tolerant to other classes of insecticides, in some countries in Europe and China its use is banned due to its high toxicological potential [2,3,4].

This prohibition, in part, is related to the problem that only 1% of the pesticides applied in agriculture reach the crops, with the rest dispersed in the environment [5]. Prado and colleagues [6] reported that only 7% of the applied fipronil remains on crops. As it leaches through the soil due to natural factors (e.g., rain, leaching and wind), the presence of fipronil and its metabolites in soil, water bodies and sediments has become alarming [2]. The indirect contact of fipronil with surfaces, such as treated clothing and animals, are also potential sources of contamination and poisoning [7]. Thus, the presence of this compound in the environment is mainly due to agricultural and domestic practices, being frequently found in different matrices varying in concentrations from µg/L to mg/L [8]. Finally, the use of compounds such as fipronil poses risks to the environment due to their long persistence, with a half-life of up to 200 days [1].

Fipronil also possesses non-specific actions, in other words, this compound is potentially toxic to non-target living organisms, mainly aquatic organisms. In this context, in the recent study by Park et al. [8], fipronil was found to be toxic to zebrafish (*Danio rerio*) embryos at 2.5 mg/L and above. In other studies, with environmentally relevant concentrations, lethality was observed for the microcrustacean *Daphnia magna* from 0.07 to 0.3 mg/L, and inhibition of the photosynthetic activity of the chlorophycean microalga *Chlamydomonas reinhardtii* at 2.4 mg/L [9]. Biochemical and genetic response modifications were also observed in organisms exposed to fipronil. For example, Monteiro et al. [10] elucidated the inhibition of antioxidant activity and of motor protein expression and globin biosynthesis in *Chironomus riparius* exposed to 0.08 µg/L for 48 h. Similarly, El-Murr et al. [11] identified an increase in liver enzymes, indicating hepatotoxicity, and showed a decrease in antioxidant activity in the Nile tilapia (*Oreochromis niloticus*) exposed to 42 µg/L fipronil for 96 h.

As mentioned earlier, fipronil and its metabolites originated during the degradation process, and are responsible for most of the contamination of agricultural fields [12]. These metabolites come from the mineralization process of the fipronil molecule, produced through its oxidation, reduction, hydrolysis and photolysis, producing fipronil-sulfone, fipronil-sulfide, fipronil-amide and fipronil-disulfenyl, respectively [1]. Due to its high harmfulness, especially of fipronil-sulfone, a metabolite even more toxic than fipronil, studies focused on the remediation of these molecules using microorganisms have become an approach widely used in order to minimize the impacts caused by environmental exposure to these compounds [2,3,6,7,13,14]. Given these approaches, biological processes are the most recommended [2] and the microorganisms selected from the environment contaminated with these compounds are capable of using fipronil as a source of carbon and nitrogen, making the biodegradation process more promising [6,15].

In this context, it is important to try to minimize the environmental impacts associated with the use of fipronil, as well as to identify microorganisms with the potential to metabolize fipronil and its derivatives. Consequently, this research aims to answer the hypothesis of whether, from fipronil-contaminated soils, we can isolate microorganisms capable of biodegrading this compound and with the potential to be used in bioremediation. For this purpose, we bioprospected and analyzed the profile of metabolites produced during the metabolization of fipronil, as well as the biodegradation kinetics of this compound, aiming to find, in contaminated soil, the microorganism with the highest capacity to degrade this important agricultural compound.

## 2. Materials and Methods

### 2.1. Isolation of Microorganisms from Soil Samples

**Figure 1:**
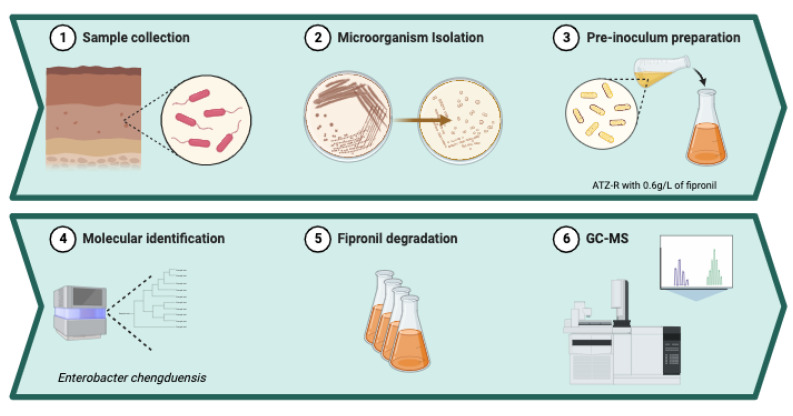
Schematic representation of the main steps carried out in this work.

In this study, microorganisms were isolated from soil samples collected at a depth of 0–10 cm in an area with a history of fipronil application, from the Experimental Farm of the Federal University of Grande Dourados, located in Dourados, MS, Brazil (22°48′53″ S and 54°44′31″ W). To initiate the isolation process, ten grams of soil were suspended in 90 mL of 0.9% saline solution, and a serial dilution (ranging from 10^−1^ to 10^−4^) was carried out. Subsequently, 100 µL of each sample was spread-plated using the solid ATZ-R method, which is a procedure previously elucidated by Prado et al. [6]. This medium was enriched with 0.6 g/L of fipronil. The plated samples were then incubated at a constant temperature of 30 °C for a 48 h. After incubation, bacterial colonies displaying distinct characteristics were selected for further analysis. These bacterial colonies underwent purification through streak plating; subsequently, a Gram stain procedure, as detailed by Prado et al. [6], was executed to ascertain the presence of spores and determine cell wall composition. For a more comprehensive understanding, the critical steps used in this work are described in detail in Figure 1.

### 2.2. Molecular Identification of Strain and Phylogenetic Tree

The strains were molecularly identified through the amplification of the 16S rDNA gene, followed by sequencing using the Sanger method. These analyses were conducted at the Centralized Multi-User Laboratory for Large-scale DNA Sequencing and Gene Expression Analysis, located at Unesp in Jaboticabal, SP, Brazil (LMSEQ). For amplification, the primers FD1(CCGAATTCGTCGACAACAGAGTTTGATCCTGGCTCAG) and RD1 (CCCGGGATCCAAGCTTAAGGAGGTGATCCAGCC) were employed [16]. Following the DNA sequencing, the assembly process was carried out using CAP3 software (CAP3 version 10, 2011), and the resulting contig was deposited in the GenBank database at https://www.ncbi.nlm.nih.gov/genbank/ (accessed on 5 August 2023) with the accession code OR365541.

The Basic Local Alignment Search Tool (BLAST) was utilized for sequence comparison against the Silva database [17,18] and GenBank database [18]. To evaluate the results between the databases (Silva and GenBank), sequence combinations with the highest similarity scores and the lowest e-values were used.

To provide further support for the results obtained in the similarity analysis, a phylogenetic tree was constructed. The evolutionary history was inferred using the Neighbor-Joining method [19]. The percentage of replicate trees in which associated taxa clustered together during the bootstrap test (1000 replicates) is presented adjacent to the branches. The evolutionary distances were computed using the p-distance method and are expressed as the number of base differences per site. The rate variation among sites was modeled with a gamma distribution (shape parameter = 1). This analysis involved 7 nucleotide sequences. All positions containing gaps and missing data were eliminated (complete deletion option). There were a total of 1299 positions in the final dataset. Evolutionary analyses were conducted using MEGA11 software (version 11) [20].

### 2.3. Pre-Inoculum Preparation

To prepare the pre-inoculum, the bacteria were cultivated in an enrichment medium containing 0.6 g/L of fipronil and 1% yeast extract. Then, the Erlenmeyer flasks were incubated at 30 °C for 48 h and orbital agitation at 100 rpm. So, the cells were washed with ATZ-R after being centrifuged for 10 min at 5 °C, 3500 rpm, and the supernatant was discarded. This washing process was repeated three times to ensure the thorough removal of any residual yeast extract. Subsequently, the cells were resuspended in ATZ-R to an optical density (OD) of 0.8–1.0 (equivalent to a concentration of 10^8^ cells/mL), as measured at a wavelength of 600 nm. These washed cells were utilized as the inoculum for the degradation experiments.

### 2.4. Fipronil Degradation

The experiments were performed as described by Prado et al. [6]. Then, bacterial growth was measured by the dry biomass for 14 days. The bacterial flasks were incubated at 30 °C with continuous agitation at 140 rpm, and samples were collected at seven defined time points (0, 3, 5, 7, 10, 12, and 14 days), as per the methodology detailed by Uniyal et al. [13]. All tests were reproduced in triplicate, with each flask containing liquid ATZ-R solution (30 mL), including fipronil at a concentration of 0.6 g/L and 1 mL of the inoculum (10^8^ cells/mL). For comparative purposes, biotic controls (without fipronil + bacterial inoculum) and abiotic controls (with fipronil + without bacterial inoculum) were established. Abiotic controls were specifically set up at the beginning (0 days) and after 14 days to assess any non-biological degradation of fipronil. Additionally, a dry biomass analysis was conducted to verify the sterility of the control cultures. Subsequently, the samples were subjected to centrifugation at 4 °C, operating at 3500 rpm for 15 min. The resulting supernatant was stored at −20 °C for subsequent GC-MS analysis, while the biomass pellet was employed for growth analysis. 

The total degradation values of the samples were determined using Equation (1), in which total degradation (TD) is calculated by dividing the difference between the initial fipronil (F*i*) and the final fipronil (F*f*) concentration, by the abiotic degradation (AD).

(1) $$TD=\frac{Fi-Ff}{AD\times 100}$$

The biological degradation rate (BD) was calculated by discounting the TD value of the AD, multiplying to 100, as follows in Equation (2).

(2) $$BD=\left(TD-AD\right)\times 100$$

The preparation of the pre-inoculum involved culturing the bacteria in liquid ATZ-R with yeast extract (1%) and fipronil (0.6 g/L). After 48 h of growth, the bacterial cells underwent a rigorous washing to ensure complete removal of residual yeast extract. Thus, the bacterial cells were subjected to centrifugation at 5 °C for 10 min, 3500 rpm. The resulting supernatant was carefully discarded, and the cells were subsequently washed three times with liquid ATZ-R containing fipronil (0.6 g/L). The washed cells were then resuspended in liquid ATZ-R to an OD reading within the range of 0.8 to 1.0 (10^8^ cells/mL), as measured at a wavelength of 600 nm. These cells were utilized as the inoculum for the subsequent degradation experiments.

### 2.5. GC-MS Conditions and Analysis of Fipronil Biodegradation and Metabolites Quantification

In this section, we describe the conditions and procedures for the GC-MS analysis of fipronil and its metabolites, using the same working conditions and characteristics established by Prado et al. [6]. Thus, were used chemicals with high-purity, such as fipronil (Chem Service, West Chester, PA, USA, 99.5% purity), fipronil-sulfone (Badische Anilin & Soda Fabrik (BASF), Ludwigshafen am Rhein Alemanha, 99.7% purity) and fipronil-sulfide (BASF, 98.8% purity). Stock standard solutions of 10 ng/µL were meticulously prepared in acetone. These working solutions served as the basis for calibration curves. The fipronil and its metabolites (fipronil-sulfide and fipronil-sulfone) was carried out using a GC-MS system (TRACE 1300 CG-MS, Thermo Fisher Scientific, Waltham, MA, USA) at the Embrapa Agopecuária Oeste Environmental Analysis Laboratory, Dourados, Brazil. The samples were subjected to extraction with ethyl:acetate in a 1:1 (*v*/*v*) ratio and homogenized for 2 min in orbital shakers. The resulting organic phase was collected and concentrated through rotary evaporation. Subsequently, it was resuspended in 4 mL of acetone. The separation of compounds was achieved using a TG-1 MS capillary column (30 m × 0.25 mm × 0.50 µm). The oven temperature program consisted of the following steps: initial temperature of 60 °C for 1 min, followed by heating to 200 °C at a rate of 30 °C/min and maintained for 5 min, further heating to 270 °C at a rate of 30 °C/min and maintained for 5 min, and finally, heating to 300 °C at a rate of 30 °C/min and maintained for 11 min. The entire run duration was 30 min. Helium served as the carrier gas at a flow rate of 1 mL/min. The injector temperature was maintained at 280 °C in a splitless injection mode, while the transfer line was kept at a temperature of 250 °C.

### 2.6. Statistical Analysis

All experiments were evaluated by analysis of variance (ANOVA). The significant difference was calculated using the Fisher mean test (*p* < 0.05) (Minitab 20.0).

## 3. Results and Discussion

### 3.1. Isolation and Molecular Identification

Seven bacteria capable of growing in an ATZ-R medium containing fipronil (0.6 g/L) as the main carbon source were isolated (results not shown) from the soil samples. After successive growth evaluations, the isolated G2.8 showed the best growth potential for 96 h, being selected for the fipronil biodegradation assays. In a preliminary analysis of the morphology of the isolate, it was observed that it is a Gram-negative rod, catalase positive and oxidase negative. The selective pressure due to the high toxicity of fipronil probably proved to be a limiting factor in obtaining more isolates from soil samples. In other studies, microorganisms capable of degrading fipronil have been isolated and studied [21,22,23]; however, many studies aimed at isolating microorganisms for subsequent biotechnological application have been limited as more than 99% of microorganisms cannot be cultured on media [24], and therefore limited knowledge about the degradation pathway of fipronil is available.

**Figure 2:**
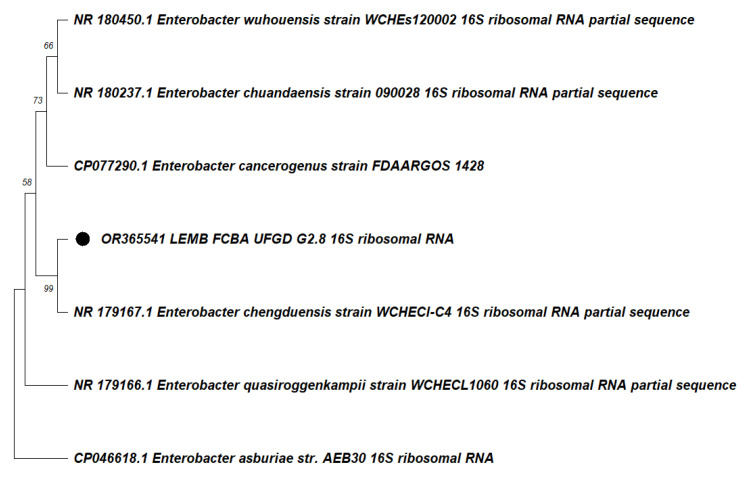
Phylogenetic tree constructed by Neighbor-joining method with 16S rRNA gene sequences of different *Enterobacter* species. The circle in the phylogenetic tree indicates the 16S rRNA sequence of the isolated bacterium.

In line with the above, once the isolate with the ability to grow with fipronil was isolated, it was molecularly identified by amplification of the 16S rDNA gene and sequenced. After sequencing and assembly of the 16S rDNA gene of isolate G2.8 OR365541, the contig was aligned with the most similar sequences in the GenBank database. Among the most similar sequences strain, G2.8 OR365541 clustered closely with *Enterobacter chengduensis* NR179167.1, supported by a bootstrap value of 99. Figure 2 shows the clustering of the DNA sequence of the 16S rRNA gene of the bacteria *E. chengduensis*.

Regarding the genus of the microorganism isolated in this work, *Enterobacter* belongs to the group of Gram-negative bacteria of the large phylum Proteobacteria. These microorganisms are widely distributed in the environment, and one of the predominant phyla has already been described in a metagenomic study with fipronil [25]. However, no studies have reported the potential of bacteria of the genus Enterobacter as fipronil degraders, only representatives of the phylum Proteobacteria. For example, Cappeline et al. [26] identified that the species *Burkholderia thailandensis* was able to degrade fipronil and its metabolites fipronil-sulfide and fipronil-sulfone. In another study, Bhatti et al. [7] identified that a non-pathogenic strain of *Escherichia coli* was able to bioaccumulate and biotransform fipronil. In the same vein, Kumar and colleagues [27] observed that soil bacteria of the phylum *Paracoccus* sp. degraded 80 µg/kg fipronil in sandy soil after 30 days. At et al. (2019) observed that fipronil degradation in field by *Staphylococcus arlettae* was 81.94% (100 mg/kg fipronil) after 30 days, while Imaniar et al. [28] identified that the species *Pseudomonas aeruginosa* was able to degrade 65% of fipronil (40 mg/L) after 3 days.

In addition, studies have shown that the genus *Enterobacter* has a high capacity for the biodegradation of toxic compounds, e.g., synthetic polymers (polyethylene and polypropylene), through the bacterial consortium of *Enterobacter* sp. and *Pseudomonas* sp. [29]; insecticide (endosulfan), with *Enterobacter asburiae* JAS5 and *Enterobacter cloacae* JAS7 [30]; azo dyes (reactive yellow 145 and reactive red 180), by the *Enterobacter hormaechei* species [31].

Regarding the species isolated in this study, the bacterium *E. chengduensis* is a new species described in 2019 in China [32,33]. This species has been reported due to its potential to metabolize lignocellulosic compounds [34]. However, no study reports its potential as a pesticide biodegradation strain. Most studies report that the species is related to human infections, and is an opportunistic pathogen normally associated with other species such as *E. asburiae*, *E. cloacae*, *E. hormaechei*, *Enterobacter kobei* and *Enterobacter ludwigii* [27,28].

### 3.2. Fipronil Degradation

The first empirical evidence supporting the involvement of microbes in fipronil degradation was provided by Zhu et al. [14]. They observed that the degradation of fipronil occurred at a significantly faster rate in non-sterile soil (half-life of approximately 9 days) compared to sterile soil (half-life of approximately 33 days). In recent decades, numerous studies have concentrated on the microbial degradation of fipronil. However, it is worth noting that up to the present time, only a limited number of microbial species have been isolated and characterized for their ability to metabolize fipronil [1,2]. Indeed, bacteria have the remarkable ability to produce enzymes involved in the degradation of fipronil through metabolic pathways that had not been previously described in the scientific literature [1]. However, it is known that autochthonous microorganisms are able to mineralize fipronil and its intermediate metabolites from the soil and water environments [2]. In this way, bioprospecting microorganisms from fipronil-contaminated environments can accelerate the biodegradation process [2,6,13,15] as the bioprospected microorganisms, which have been able to grow in the presence of these compounds, should have the enzymatic machinery necessary for the metabolization of fipronil.

As mentioned above, the fipronil contamination of soils can alter the diversity of microbial communities [9,35,36]. This is because the high toxicity of fipronil and the relatively low water activity found in soils probably act as a selective pressure for the growth of microorganisms; in other words, only those microorganisms possessing the enzymatic machinery that enable them to degrade these compounds are able to survive. An example of this has been observed in the recently published study by Guima et al. [20], where the authors observed a variation in the microbial community in the presence of fipronil; furthermore, the authors have shown that the phyla represented by Proteobacteria, Actinobacteria and Firmicutes have been benefited in this condition. Additionally, some genera of bacteria may also possess the capacity for chemotaxis, which helps them to identify the presence of contaminants through chemical signals, as is the case with actinobacteria and diazotrophic bacteria, helping them to survive in contaminated environments [1].

In line with the previous work, once the bacteria that showed the best growth in the presence of fipronil had been bioprospected and identified here, their ability to degrade this compound was determined. To do this, firstly, the growth kinetics of *E. chengduensis* was evaluated by assessing the dry biomass. Table 1 shows that the highest growth rate obtained by *E. chengduensis* occurred on day 7. Furthermore, no microbial growth was observed in the biotic and abiotic controls, showing that *E. chengduensis* was dependent on and grew exclusively using fipronil as an energy source.

**Figure 3:**
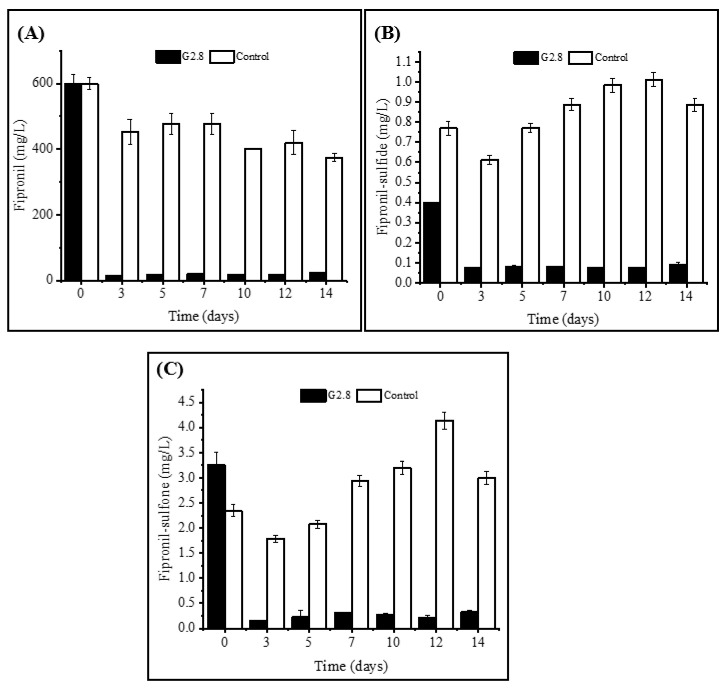
Profile of fipronil biodegradation and metabolite production by isolate G2.8. (**A**) fipronil, (**B**) fipronil-sulfone and (**C**) fipronil-sulfide. Statistics analyses by Fisher’s test (*p* < 0.05) presented as mean ± standard deviation.

Secondly, the degradation taxa of fipronil and its derivatives were quantified by GS-MS. In this regard, it was observed that after 14 days, *E. chengduensis* was able to degrade 96% of the fipronil present in the sample (Figure 3A), which highlighted the biodegradation capacity of the bioprospected bacteria. On the other hand, it was observed that the highest degradation rate occurred between the first and third day of the experiment (Figure 3A). Simultaneously, it was identified that the highest biomass increase occurred after 5 days (Table 1). Furthermore, it was also found that 40% up to 45% of the degradation occurring was due to environmental factors (chemical degradation, photodegradation, adsorption, among other possible processes), as observed by the decrease in the abiotic control values (Figure 3A). Finally, it was observed that there was a 50% reduction from the initially inoculated biomass to the final time, demonstrating that *Enterobacter chengduensis* was exclusively dependent on the presence of fipronil for its growth (Table 1).

Similar results were obtained in the study by Viana et al. [3], where the authors observed that the *Bacillus amyloliquefaciens* strain RFD1C was able to degrade 93% of fipronil (10 mg/L) over a period of 5 days. Similarly, Uniyal et al. [13] identified that a strain of *Stenotrophomonas acidaminiphyla* was able to degrade 70% of fipronil (50 mg/L) in 14 days. In another study, Bhatt and colleagues [15] demonstrated the ability to degrade 76% of fipronil (50 mg/L) by the *Bacillus* sp. strain FA3 over 15 days. These results are also in line with those obtained by Gangola et al. [37], who observed a 93% degradation rate of fipronil (450 mg/L) after 15 days of growth by the *Bacillus* sp. strain 3C. In another similar study, Abraham et al. [38] also obtained an 100% degradation rate of fipronil (500 mg/L) after 15 days by the *Streptomyces rochei* strain AJAG7. Finally, some studies have also demonstrated the ability of fungi such as *Trametes versicolor* [39] and *Aspergillus glaucus* AJAG1 [40] to degrade fipronil. Taken together, these findings suggest that microorganisms isolated from fipronil-contaminated environments show good performance in the biodegradation process of this compound, which could be a promising tool for the decontamination of fipronil-contaminated agricultural environments. These findings collectively underscore the crucial contribution of microorganisms in the biodegradation of fipronil and its metabolites.

Regarding the production of metabolites during the fipronil degradation process, over 14 days, a higher production of the metabolite fipronil-sulfone produced by the fipronil oxidation pathway was observed (Figure 3B) in relation to the fipronil-sulfide produced by the reduction in this compound (Figure 3C). On the other hand, it was observed, by comparison with the abiotic control, that fipronil-sulfone showed a 25% degradation, while fipronil-sulfide showed a 17% degradation in 14 days. Both metabolites showed an increase at 5 days (Figure 3B,C) and degradation at 12 days. These data suggest that the presence of *Enterobacter chengduensis* could influence the biodegradation process of fipronil, being responsible for the simultaneous production and degradation of metabolites in an accelerated manner.

The observed increase in fipronil-sulphone production by *Enterobacter chengduensis* could be related to biomass decomposition (Table 1). Another factor that could be related is that the microorganism possesses the active oxidase enzyme (see isolation and molecular identification), presenting an increased oxidation capacity. Little is known about the mechanisms related to the pathways and metabolization process of fipronil. The observations of Bhatt and colleagues [2] further support the hypothesis that aerobic microorganisms with active oxidase, such as the *Enterobacter chengduensis* biosprospected here, have a higher capacity to oxidize fipronil to generate fipronil-sulfone, while microaerophiles or anaerobes further reduce fipronil, forming fipronil-sulfide.

Finally, although the metabolic pathways of fipronil have not been described, it is known that its metabolism occurs through biochemical processes. Specifically, it has been described that the detoxification of this molecule occurs through mechanisms related to the cytochrome P450 complex, divided into two stages, I and II [41]. In step I, oxidation of the pyrazole ring of fipronil occurs. Step II occurs through detoxification by catalytic enzymes, which first promote hydroxylation of the aromatic ring (target position), with consequent glycosylation of the added hydroxyl [1]. Even without the exact identification of the fipronil degradation pathways, some P450 enzymes (example: CYP18, CYP302, CYP4 and CYP6) have been identified as fipronil detoxification enzyme genes [22]. In this sense, the findings obtained within this study motivate future research and searches for microorganisms with potential to degrade fipronil like the one described here, as well as to understand the metabolic mechanisms used by them and, thus, use this information for the development of new tools, such as bacterial consortia, to try to minimize the impacts of human activity on agricultural soil.

## 4. Conclusions

In this work, from soils contaminated with fipronil, we have been able to isolate microorganisms capable of biodegrading this compound and with the potential to be used in bioremediation. Therefore, the hypothesis initially proposed was confirmed.

In this sense, from the data obtained, it can be affirmed that the microorganisms present in the soil contaminated by fipronil can attribute expressive results in the degradation of fipronil. It was identified that the *Enterobacter chengduensis* strain, isolated from a maize crop soil contaminated by fipronil, showed a high fipronil degradation capacity in a relatively short period of time. Probably, this activity is observed due to its prolonged exposure to the pesticide. Moreover, it was observed that the metabolites produced during fipronil degradation did not present toxicity to the microorganisms, thus allowing their functional metabolism during the mineralization process.

Consequently, *Enterobacter chengduensis* could be a good candidate to contribute to environmental protection, being used in bioremediation processes in areas contaminated with fipronil, minimizing the negative impacts of human intervention in the environment.

## Figures and Tables

**Table 1:** Growth kinetics of *Enterobacter chengduensis.*

Time (Days)	Dry Biomass (g/L)		
	G2.8	Biotic Control	Abiotic Control
0	0.590 ± 0.13	0.037 ± 0.01	NBG
3	0.668 ± 0.12	-	-
5	0.571 ± 0.05	-	-
7	0.803 ± 0.07	-	-
10	0.706 ± 0.08	-	-
12	0.672 ± 0.10	-	-
14	0.576 ± 0.06	0.02 ± 0.00	NBG

Statistics analyzed by Fisher’s test (*p* < 0.05) presented with mean ± standard deviation. Biotic and abiotic control were performed only at the beginning and at the end of the trials; NBG: no bacterial growth.

## Data Availability

The contig was deposited in the GenBank database at https://www.ncbi.nlm.nih.gov/genbank/ with the accession code OR365541 (accessed on 5 august 2023).
